# A Semisupervised Support Vector Machines Algorithm for BCI Systems

**DOI:** 10.1155/2007/94397

**Published:** 2007-07-25

**Authors:** Jianzhao Qin, Yuanqing Li, Wei Sun

**Affiliations:** ^1^Institute of Automation Science and Engineering, South China University of Technology, Guangzhou 510640, China; ^2^ Shenzhen Institute of Advanced Integration Technology, Chinese Academy of Sciences, The Chinese University of Hong Kong, Hong Kong; ^3^ School of Maths, Central-South University, Changsha 410008, China

## Abstract

As an emerging technology, brain-computer interfaces (BCIs) bring us new communication interfaces which translate brain activities into control signals for devices like computers, robots, and so forth. In this study, we propose a semisupervised support vector machine (SVM) algorithm for brain-computer interface (BCI) systems, aiming at reducing the time-consuming training process. In this algorithm, we apply a semisupervised SVM for translating the features extracted from the electrical recordings of brain into control signals. This SVM classifier is built from a small labeled data set and a large unlabeled data set. Meanwhile, to reduce the time for training semisupervised SVM, we propose a batch-mode incremental learning method, which can also be easily applied to the online BCI systems. Additionally, it is suggested in many studies that common spatial pattern (CSP) is very effective in discriminating two different brain states. However, CSP needs a sufficient labeled data set. In order to overcome the drawback of CSP, we suggest a two-stage feature extraction method for the semisupervised learning algorithm. We apply our algorithm to two BCI experimental data sets. The offline data analysis results demonstrate the effectiveness of our algorithm.

## 1. INTRODUCTION

A brain-computer interface is a communication system that does not depend on brain's normal output pathways of peripheral nerves and muscles. It provides a new augmentative communication technology to those who are paralyzed or have other severe movement deficits [1].

For many BCI systems, a tedious and time-consuming training process is needed to train the user and system parameters, for example, the parameters of the translation algorithm. In BCI competition III, reducing the training process has been explicitly proposed as a task by Schalk [2].

In this paper, we resort to semisupervised learning to train an SVM classifier. Compared with the case of supervised learning, semisupervised learning can build better classifiers by using large amounts of unlabeled data, when the labeled data are expensive or time consuming to obtain [3, 4] (in BCI systems, the training process can be taken as the labeling process). Thus, the performance of semisupervised learning can still be satisfactory. The semisupervised learning algorithms that have been developed so far include EM algorithm [5], self-training algorithm [6], cotraining algorithm [7], graph-based methods [8, 9], and so forth. A survey of semisupervised learning can be found in [3, 4].

In [10], Bennett and Demiriz proposed a semisupervised SVM. Given a set of labeled data and a set of unlabeled data, a semisupervised SVM was trained using both the labeled data and unlabeled data. This algorithm can be implemented using mixed integer programming. However, since the computational burden of mixed integer programming will increase greatly with the number of integer variables (i.e., the number of unlabeled samples), it is unacceptable when the size of the unlabeled data set is large, especially when we apply this algorithm to the online BCI system. Thus, we propose a batch-mode incremental training method for the semisupervised SVM.

There are two basic ideas in this method: (1) we assume that the users' electroencephalography (EEG) change gradually during the use of BCI systems. Therefore we can decompose the unlabeled data set into several subsets; then we use mixed integer programming to adjust the parameters of the semisupervised SVM incrementally with the entering of each subset, that is, we do mixed integer programming only based on a small-scale unlabeled data set each time. (2) For each unlabeled subset, we first select and label the most reliable data; then do the mixed integer programming based on the remaining unlabeled data set. This can further reduce the number of integer variables (i.e., the number of unlabeled data) for each running of the mixed integer programming.

Additionally, in BCI systems, the common spatial patterns (CSP) method is very effective for extracting features from the EEG recordings [11–13] (we refer to the feature extracted by CSP method as “CSP feature” in this paper). The extraction of the CSP feature is label dependent, that is, the CSP feature should be extracted from the labeled data set. If the number of labeled samples is too small, the transformation matrix for CSP feature extraction cannot be estimated accurately. This will result in an ineffective CSP feature. In order to overcome the drawback of CSP feature, we suggest a two-stage feature extraction method, that is, we first extract a dynamic power feature and perform an initial classification on a part of the unlabeled data at the first stage (the first several loops) of our semisupervised learning algorithm. Next, we extend the small labeled data set by including the most confidently classified unlabeled data with the predicted labels. Based on the extended labeled data set, somewhat reliable CSP features and better classification result can be obtained at the second stage (the remaining loops) of our semisupervised learning algorithm.

We evaluate the semisupervised SVM algorithm using a data set from an EEG-based cursor control experiment carried out in Wadsworth Center [14] and a data set from a movement imagination experiment provided by Department of Computer Engineering University of Tübingen, Germany, and Institute of Medical Psychology and Behavioral Neurobiology [15]. Data analysis results demonstrate the validity of our algorithm.

The organization of this paper is as follows. In Section 2, we introduce the proposed methods, including feature extraction, semisupervised SVM, and batch-mode incremental training. Section 3 presents the data analysis results. In Section 4, we discuss our algorithm in detail.

## 2. METHODS

In this section, we first present the dynamic CSP feature extraction and the dynamic power feature extraction method; then we combine them to form a two-stage feature extraction method. Next, we introduce the semisupervised SVM algorithm. Finally we present the batch-mode incremental learning method for training the semisupervised SVM.

### 2.1. Dynamic CSP feature extraction

The common spatial patterns (CSP) is a method that has been applied to EEG analysis to classify the normal versus abnormal EEGs [16] and find spatial structures of event-related (de-)synchronization [12]. We define two CSP feature in this paper: (1) nonnormalized CSP feature, (2) normalized CSP feature. The nonnormalized CSP feature is extracted directly from the covariance matrix of the raw or filtered EEG signal. The normalized CSP feature is extracted from the normalized covariance matrix of the raw or filtered EEG signal. The advantages of the nonnormalized CSP feature are: (1) it keeps the amplitude information of the EEG signal; (2) its dimension is usually half of the normalized CSP feature. The normalized CSP feature also has its advantages. It can reduce the influence of the scaling (due to the change of the electrode impedances or other causes) of the amplitude of the recorded EEG.

Now, we present the extraction of the dynamic nonnormalized CSP feature, which is similar to the method described in [11]. First, we filter the raw EEG in *μ* rhythm frequency band. The following CSP feature extraction is based on the filtered signals. In order to reflect the change of brain signals during a trial, we extract a dynamic CSP feature, that is, we separate the time interval of each trial into *f* overlapped time segments. For each time segment, we calculate a CSP feature vector as follows. The CSP analysis in the *i*th (*i* = 1,…,*f*) time segment involves calculating a matrix W_*i*_ and diagonal matrix D_*i*_ through a joint diagonalization method as (1):

(1) $$W_{i}Z_{i}^{n}W_{i}^{T}=D_{i},\;\;W_{i}Z_{i}^{m}W_{i}^{T}=1-D_{i},$$

where **Z** ^*n*^_*i*_ and **Z** ^*m*^_*i*_ are covariance matrices of EEG data matrices **E** ^*n*^_*i*_ and **E** ^*m*^_*i*_ (one row of the EEG data matrices corresponds to one channel EEG signal). *n*and*m* denote two different classes (for the cursor control experiment, *n*and*m* represent two different targets; for the movement imagination experiment, *n*and*m* denote two different movement imaginations). Using all trials with class *n*, we construct the matrix **E** ^*n*^_*i*_ by trial-concatenating the filtered EEG data in the *i*th time segments of every trial. **E** ^*m*^_*i*_ is obtained similarly except that it corresponds to the trials with class *m*. The diagonal elements of D_*i*_ are sorted with a decreasing order.

After obtaining the transformation matrix W*_i_*, we now extract CSP feature in the *i*th time segment of a trial (*i* = 1,…,f). We first calculate a covariance matrix using the filtered EEG signals in the *i* th time segment; then we take the first *p* or the last *p* main diagonal elements of the transformed (by W*_i_*) covariance matrix. Note that the first *p* diagonal elements correspond to *p* largest eigenvalues in the diagonal matrix D*_i_* above, the last *p* smallest eigenvalues. Thus we obtain a *p* -dimensional CSP feature for each time segment. We concatenate the CSP features of *f* time segments to construct the *p*⋅*f* -dimensional dynamic CSP feature of each trial, which is denoted as CF = [CF_1_, CF_2_, …,CF_*f*_].

The normalized CSP feature [12] is almost the same as the above CSP feature, except that: (1) the correlation matrix is normalized by dividing the trace of the correlation matrix; (2) the first *p* and the last *p* main diagonal elements of the transformed covariance matrix are taken, then normalized by dividing the sum of the 2*p* elements followed by a log-transformation. The log transformation serves to approximate normal distribution of the data [12]. Thus the dynamic normalized CSP feature for *f* time segments is 2*p*⋅ *f* -dimensional.

### 2.2. Dynamic power feature extraction and the two-stage feature extraction method

According to the above definition of CSP feature, it is obvious that the CSP feature extraction is dependent on the labels of the trials in the training set. If the number of labeled samples is too small, the transformation matrix of W*_i_* cannot be estimated accurately, sometimes, even poorly. This will result in an ineffective CSP feature. In this subsection, we solve this problem by combining the power feature with the CSP feature to form a two-stage feature extraction method.

Our method is based on the following two facts: (1) the power feature extraction is not so dependent on sufficient labeled data set as CSP feature extraction; (2) the power feature is less powerful than CSP feature when the training data is sufficient. Thus, in the first several loops of our semisupervised algorithm (the first stage), we use power feature to obtain an initial classification on a part of the unlabeled data set. Based on the initial classification result, we perform CSP feature extraction and classification in the later loops of our semisupervised algorithm (the second stage).

The power feature extraction is as follows: we first calculate the power values of selected EEG channels in the μ frequency band for each time segment. Then, we scale the power values, that is, the power value of each selected channel is divided by the sum of the average power values of the two different classes of this channel (the average power values are calculated from the labeled data set).

For each time segment, we choose 2 channels which are the most discriminant for the power feature extraction. We denote the power feature for time segment *i* as *i* as PF*_i_*=[PF*_ij_*, PF*_ij_*], *i* = 1,…,*f*, *j* = 1,2 (*f* is the number of time segments); then concatenate the power values of all the time segments of a trial to form the dynamic power feature of a trial, which is denoted as PF = [PF_1_, PF_2_, …,PF_*f*_].

For each time segment, the selection of channels is dependent on the discriminant ability of the power feature of the channels. The discriminant ability of the power feature of each channel is calculated as follows:

(2) $$\begin{array}{ll} {\text{FR}}_{ij}={\left(\text{mean}({\text{PF}}_{ij}^{n})-\text{mean}({\text{PF}}_{ij}^{m})\right)}^{2} & \;i=1,\ldots ,f,\\ & \;j=1,\ldots ,h, \end{array}$$

where *i* denotes the *i*th time segment, and *j* denotes the *j*th channel; *f* is the number of time segments; *h* is the number of channels; *n* and *m* represent two different classes. The bigger the value of FR *_ij_* is, the stronger discriminant ability of the power feature for channel *j* and time segment *i*is.

At the first stage (first several loops of semisupervised learning), we only extract the above dynamic power feature from the trials. After these loops of semisupervised learning, a part of the unlabeled data set is classified. Then, at the second stage, using the most confidently classified data with predicted labels to extend the given small training data set, we extract somewhat reliable dynamical CSP feature and perform the later loops of semisupervised learning. The detailed procedure of our feature extraction method with the batch-mode incremental training method is presented in Section 2.4

### 2.3. Semisupervised SVM

In this subsection, we review the semisupervised SVM introduced by Bennett and Demiriz [10].

Given a training set of labeled data

(3) $$\{(x_{i},y_{i})|(x_{i},y_{i})\in R^{n}\times \{\pm 1\},\;i=1,\ldots ,\ell \},$$

where x_1_, …,x_*e*_ are the *n* dimensional features that have been labeled as *y*_1_, …,*y*_e_; and a set of unlabeled data

(4) $$\{x_{i}|x_{i}\in R^{n},\;i=\ell +1,\ldots ,\ell +k\},$$

in [10], a semisupervised SVM was defined as

(5) $$\begin{array}{c} \underset{w,\text{b},\eta ,\xi ,\delta }{\min}C[\overset{\ell }{\underset{i=1}{\sum }}\eta_{i}+\overset{\ell +k}{\underset{j=\ell +1}{\sum }}\min(\xi_{j},\delta_{j})]+{||w||}_{1},\\ \text{s}\text{.t}\text{.}\;\;y_{i}(w\cdot x_{i}-b)+\eta_{i}\geq 1,\;\eta_{i}\geq 0,\;\;i=1,\ldots ,\ell ,\\ w\cdot x_{j}-b+\xi_{j}\geq 1,\;\xi_{j}\geq 0,\;\;j=\ell +1,\ldots ,\ell +k,\\ -(w\cdot x_{j}-b)+\delta_{j}\geq 1,\;\delta_{j}\geq 0, \end{array}$$

where *C* > 0 is a penalty parameter, and η_i_, ξ_j_, δ_j_ are the slack variables that present the classification error of x_*i*_ or x_*j*_.

The semisupervised SVM can be reformulated as follows:

(6) $$\begin{array}{c} \underset{w,b,\eta ,\xi ,\delta ,d}{\min}C[\sum_{i=1}^{\ell }\eta_{i}+\sum_{j=\ell +1}^{\ell +k}(\xi_{j}+\delta_{j})]+∥w{||w||}_{1},\\ \text{s}\text{.t}\text{.}y_{i}(w\cdot x_{i}-b)+\eta_{i}\geq 1,\eta_{i}\geq 0,\;i=1,\ldots ,\ell ,\\ w\cdot x_{j}\text{​}-\text{​}b\text{​}+\text{​}\xi_{j}\text{​}+\text{​}M(1-d_{j})\geq 1,\xi_{j}\geq 0,\;j\text{​}=\ell +1,\ldots ,\ell +k,\\ -(w\cdot x_{j}-b)+\delta_{j}+Md_{j}\geq 1,\delta_{j}\geq 0,\;d_{j}=\{0,1\}, \end{array}$$

where *d_j_* is a decision variable. For each point x_*j*_ in the unlabeled data set, *d_j_* =1 means that the point is in class 1, otherwise the point is in class −1. *M*>0 is a sufficiently large constant. Mixed integer programming can be used to solve this problem. But, mixed integer programming problems are NP-hard to solve [17], even when restricted to 0-1 programs [18]. If the number of the integer variables is large, the computational burden will be very heavy. In practice, since we often encounter large amounts of unlabeled data, we should assign large amounts of integer variables for these unlabeled data. Thus, if we solve this problem using the mixed integer programming directly, the training time of semisupervised SVM is unacceptable.

### 2.4. Batch-mode incremental training method

In this section, we extend the semisupervised SVM in [10].

We divide the original unlabeled data set into several subsets, and mark them as *B*_1_, *B*_2_, …,*B*_*n*_. Each time, we do the mixed integer programming based on a subset. It is reasonable to assume that the users' EEGs change gradually during the use of the BCI systems. Using the incremental training method, the parameters of the SVM can be adjusted gradually with the entering of these several subsets (the new entered subsets represent the changed status of the users' EEG). Additionally, in order to further reduce the number of integer variables for each running of mixed integer programming, when a new subset is added, we first temporarily label the unlabeled elements in this subset using the SVM which has been trained in the previous loop; then we choose the most confidently classified elements and add them, together with their predicted labels, to the training set; finally, we use the remaining unlabeled elements for mixed integer programming. The most confidently classified elements can be determined according to the distance between the element and the separating boundary. The criteria can be formulated as follows:

(7) |x⋅w−b|≥L,

where constant *L*>0 is the distance threshold. If the distance between the element and the separating boundary is larger than *L*, we take it as a confident element.

The outline of the batch-mode incremental training method is as follows.

*Algorithm outline.* Given two data sets: a labeled data set *D_l_*, and an unlabeled data set *D_u_*.

**Step 1**

Equally divide the unlabeled data set *D_u_* into *n* subsets *B_1_*, *B_2_*, . . . , *B_n_* (for the online condition, these subsets can be collected at several time intervals); then take *B_l_* as the initial training set *T* and let *i *= 1 (*i* denotes the ith loop of the algorithm); next extract dynamic power feature from data set *T* and *B_1_* (see Section 2.2).

**Step 2**

Train an initial SVM classifier using the dynamic power features in *T* .

**Step 3**

Estimate the labels of *B_i_* using the current classifier, then choose the most confidently classified elements using (7) and add them together with their predicted labels (we mark the most confidently classified elements with their predicted labels as set *R*), to the training set *T*, that is, *T* = *T* ∪ *R*; next we denote the remaining elements of *B_i_* as *Q_i_*.

**Step 4**

Run the mixed integer programming based on *T* and *Q_i_* to get the labels of *Q_i_* and adjust the parameters of the SVM classifier; then add *Q_i_* with predicted labels to *T*, that is, *T* = *T* ∪ *Q_i_*.

**Step 5**

*i* = *i* + 1, if i is smaller than or equals to *M* which denotes the number of steps for dynamic power feature extraction, extract dynamic power features from *B_i_*; otherwise, extract dynamic CSP features from *B_i_*. Note that, for each loop, the transformation matrix of the dynamic CSP feature should be estimated again from the most confidently labeled elements of *T*; then use the new transformationmatrix to extract the dynamic CSP features from *T* again. Finally train a new SVM classifier based on the updated CSP features and their labels of *T*.

**Step 6**

If *i* equals *n*, terminate; otherwise, go back to Step 3.

Additionally, since the size of the training set is enlarged during the training procedure, the penalty parameter *C* of the semisupervised SVM should adapt to this change. Thus, we extend the empirical formula for *C* introduced in [10] as:

(8) $$C_{i}=\frac{\left(1-\lambda \right)}{\lambda (\ell_{i}+k_{i})},$$

where *i* denotes the *i*th loop. ℓ_*i*_ is the size of training set of the *i*th loop. *K_i_* is the size of unlabeled data set. We set λ = 0.01 in our following experimental data analysis.

**Figure 1:**
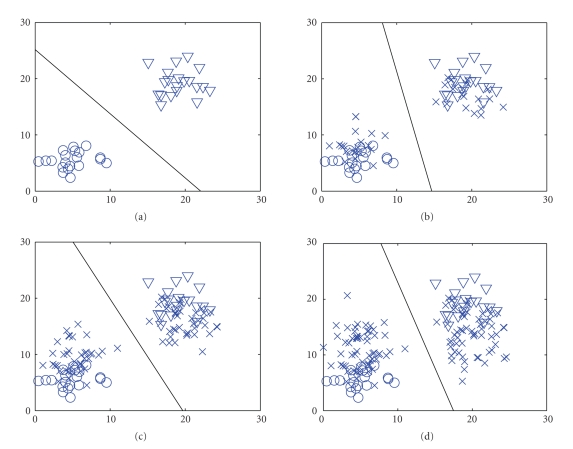
The demo of the batch-mode incremental training method (the circles and the triangles denote the labeled training samples of two classes. The crosses denote the unlabeled samples. The lines denote the separating boundary of the SVM classifier).

Figure 1 shows a demo of the batch-mode incremental training method. The circles and the triangles denote the labeled training samples of two classes. The crosses denote the unlabeled samples. The solid line denotes the separating boundary of the SVM classifier. From Figures 1(a) – (d), the unlabeled samples were added gradually. The figure shows that the separating boundary of the SVM classifier was adjusted gradually according to the entering of the unlabeled samples.

## 3. EXPERIMENTAL DATA ANALYSIS

In this section, we evaluated the semisupervised SVM-based algorithm using the data set from an EEG-based cursor control experiment and an ECoG-based movement imagination experiment. The hardware and software environments of our data analysis are as follows.

Hardware: personal computer (CPU: Intel P4 1.7 Ghz; RAM: SDRAM 512 MB).

Software: operating system: Windows 2000 professional. The main program was coded by MATLAB 6.5. The mixed integer programming problem and 1-norm SVM were solved by a free software LP-solve 2.0 C library by Michel Berkelaar and Jeroen Dirks. We repacked this software and compiled it as the mex file which can be called by MATLAB. We used the commands “cputime” to calculate the CPU time needed for training the semisupervised SVM.

### 3.1. Data analysis of an EEG-based cursor control experiment

The EEG-based cursor control experiment was carried out in Wadsworth Center. In this experiment, the subjects sat in a reclining chair facing a video screen and was asked to remain motionless during performance. The subjects used μ or β rhythm amplitude to control vertical position of a target located at the right edge of the video screen. The data set was recorded from three subjects (AA, BB, CC). Each subject's data included 10 sessions. The data set and the details of this experiment are available at http://www.ida.first.fraunhofer.de/projects-/bci/competition. For convenience, only the trials with the targets who are at the highest and lowest position of the right edge of the screen were used in our offline analysis (96 ∗ 10 trials for each subject).

To evaluate our proposed algorithm, we separated all the trials into three sets, that is, labeled data set, unlabeled data set, and independent test set. Labeled data set consists of 48 trials (about 10% of all labeled and unlabeled data) (24 trials for each target) from session 1. Unlabeled data set consists of 528 trials from the remaining trials of session 1 and all the trials of sessions 2–6; and the independent test set is composed of 384 trials of sessions 7–10. When implementing the batch-mode training method, we divided the unlabeled set into 9 subsets (each of the first 8 subset has 60 elements, and the 9th subset has 48 elements).^1^

In the data analysis, for the first two loops of our algorithm, we extracted five-time-segment dynamic power feature; then for the following loops, we extracted five-time-segments nonnormalized dynamic CSP feature from the 64-channel band pass filtered (11–14 Hz) raw EEG signal.^2^ Based on the cross-validation results obtained from the training set, we find that the first 2 main diagonal elements were more significant for discriminating for subjects AA, CC and the last 2 main diagonal elements were more significant for subject BB. Therefore, in each time segment, the first 2 main diagonal elements for subject AA, CC and the last 2 main diagonal elements for subject BB were taken as the CSP feature. The dynamic CSP feature is of 10 dimensions.

We present our data analysis results as follows. We applied our algorithm to the independent test set. By comparing the predicted target position for each trial with the true target position, the accuracy rate is obtained. The accuracy rates for the three subjects are shown in the second row of Table 1.

To further demonstrate the validity of our algorithm (Case 1), we do the following comparison.

**Case 1**

The proposed semisupervised SVM trained from labeled and unlabeled data is used to classify the independent test set.

**Case 2**

A standard 1-norm SVM trained from the labeled data is used to classify the independent test set. Note that all 0the features extracted are the dynamic CSP features in this case.

**Case 3**

The true labels of the unlabeled data are assigned; then we use these data with the original labeled data to train a standard 1-norm SVM to classify the independent test set. Note that all the features extracted are the dynamic CSP features in this case.

**Case 4**

The original training method of semisupervised SVM introduced in [10] is used to replace the batch-mode incremental training method. Note that in this case, due to the heavy computational burden, we had run the mixed integer programming for more than 24 hours, but failed to get a result. So, the accuracy in Table 1 for Case 4 is empty.

**Case 5**

A full bayes classifier-based self-training algorithm is used to replace the semisupervised SVM-based algorithm. Note that in this case, all the features extracted are the dynamic CSP features.

**Case 6**

A full bayes classifier trained from the labeled data is used to classify the independent test set. Note that in this case, all the features extracted are the dynamic CSP features.

Table 1 shows the accuracy rates for the three subjects in Cases 1, 2, 3, 5, 6. It shows that our algorithm improves the accuracy rate significantly (by 14.34%), compared with the accuracy rates obtained in Case 2. Furthermore, compared with the accuracy rate of Case 3 in which all the data (including labeled and unlabeled data) were labeled, the accuracy rate (when only 10% data were labeled) obtained by using our algorithm is only lower than it by 3.25%. From the results of Cases 5, 6, we find that except for subject AA the full bayes classifier based self-training algorithm fails to improve the accuracy rate by using the unlabeled data. In most cases, when the number of labeled samples for training full bayes classifier is small, the estimation of the parameters of the full bayes classifier is often poor. Thus, when we use this classifier trained from the labeled data to predict the classes of the unlabeled data, only very small part of the unlabeled data can be classified correctly. When only very small part of correct classified unlabeled data is available, we cannot employ the information provided by the unlabeled data. This results in the poor performance in data sets BB and CC. In some rare cases, however, the distribution of the labeled small samples also can present partial distribution of the real data, that is, the labeled samples distribute on several representative places of the real data distribution. The bayes classifier trained from the labeled data can correctly classify part of the unlabeled data. The classification accuracy is better than the accuracy in the normal case. Therefore, for data set AA, the information embedded in the unlabeled data can be used to improve the performance of the classifier by the self-training algorithm.

Table 2 lists the CPU times of training the semisupervised SVM in Cases 1, 4 for 3 different subjects. Note that the values of the CPU time are the mean of five times running of the corresponding algorithm. In Case 4, we had run our program for more than 24 hours (86400 seconds) without getting a result. It shows that the batch-mode incremental training method is much faster than the method used in Case 4.

**Figure 2:**
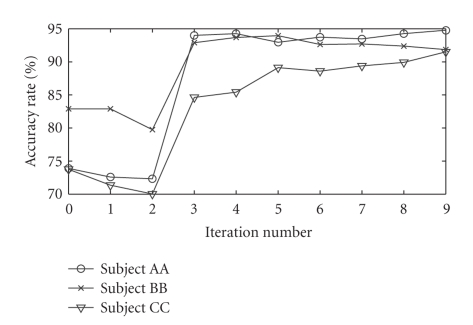
The change of the accuracy rate of the independent test set with the batch-mode incremental training process for the three subjects in the data analysis of an EEG-based cursor control experiment.

Figure 2 shows the change of the accuracy rate of the independent test set with the batch-mode incremental training process for the three subjects. It illustrates that the main trend of the accuracy rate of the independent test set increases along with the entering of the unlabeled data.

### 3.2. Data analysis of an ECoG-based movement imagination experiment

The data set of an ECoG-based movement imagination experiment was provided by Department of Computer Engineering University of Tübingen, Germany, (Prof. Rosenstiel) and Institute of Medical Psychology and Behavioral Neurobiology (Niels Birbaumer), and Max-Planck-Institute for Biological Cybernetics, Tübingen, Germany (Bernhard Schölkopf), Department of Epileptology and Universität Bonn, Germany, (Prof. Elger) [15]; and is included by BCIcompetition III as data set I. This data set and its detailed description are available at http://www.ida.first.fraun-hofer.de/projects/bci/competitioniii. During the BCI experiment, a subject had to perform imagined movements of either the remaining small finger or the tongue. The time series of the electrical brain activity was picked up during these trials using an 8 × 8 ECoG platinum electrode grid which was placed on the contralateral (right) motor cortex. The grid was assumed to cover the right motor cortex completely, but due to its size (approx. 8 × 8 cm) it partly covered also surrounding cortex areas. All recordings were performed with a sampling rate of 1000 Hz. After amplification, the recorded potentials were stored as microvolt values. Every trial consisted of either an imagined tongue or an imagined finger movement and was recorded for 3-second duration. To avoid visually evoked potentials being reflected by the data, the recording intervals started 0.5 seconds after the visual cue had ended. 278 trials were recorded in the same day which were taken as the training set in the competition. About 1 week later, 100 trials were recorded which were taken as the test set in the competition.

We took 28 trials (about 10% of all labeled and unlabeled data) from the 278 trials of training set as the labeled data set; and we took the remaining 250 trials of training set as the unlabeled data set; then took the 100 trials of test set as the independent test set. We first downsampled the original signals from 1000 Hz to 250 Hz for reducing the computational burden. In the first two loops of our algorithm, we extract five-time-segments dynamic power feature from the trials; in the remaining loops, we extract the 5-time-segments normalized dynamic CSP feature from the common average referenced (CAR) [19] and band-pass (8–12 Hz) filtered 32-channel (we chose 1 out of 2 original channels, that is, with channel numbers 2, 4, 8, …, 64) EEG data. In each time segment, the first 2 main diagonal elements and the last 2 main diagonal elements were taken as the CSP feature. The dimension of the dynamic CSP feature is 20. Note that the transformation matrix of the CSP feature of each time segment is calculated from the labeled data. We divided the unlabeled data into 4 batches. Each of the first 3 batches contains 63 elements. The fourth batch contains 61 elements.

We consider 6 cases as in EEG-based cursor control experiment data analysis.

Table 3 shows the accuracy rates in Cases 1, 2, 3, 5, 6. It shows that semisupervised learning improves the accuracy rate significantly (by 17%), compared with the accuracy rates obtained in Case 2. And, compared with the accuracy rate of Case 3 in which all the data (including labeled and unlabeled data) were labeled, the accuracy rate (when only 10% data was labeled) is only lower by 1%. From the results of Cases 5, 6, we see that bayes classifier-based self-training algorithm fails to improve the accuracy rate. In Case 4, we have run our algorithm for more than 24 hours without getting a result.

Table 4 lists the CPU time for training the SVM. Note that it is the average CPU time of five times running of the algorithm. The result also shows that the batch-mode incremental training method is much faster than the method used in Case 4.

**Figure 3:**
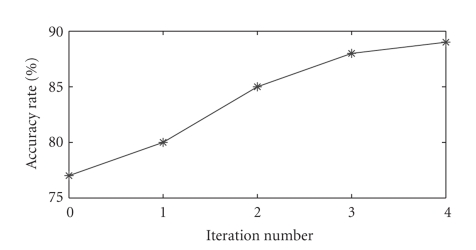
The change of the accuracy rate of the independent test set with the batch-mode incremental training process in the data analysis of an ECoG-based movement imagination experiment.

Figure 3 shows the change of the accuracy rate of the independent test set with the batch-mode incremental training process. It illustrates that the main trend of the accuracy rate of the independent set increases along with the entering of the unlabeled data.

## 4. CONCLUDING REMARKS

In this paper, we present a semisupervised SVM algorithm for BCI systems, aiming at reducing the tedious and time-consuming training process.

The advantages of our algorithms are as follows.

1. It achieves a satisfactory generalization performance by using the unlabeled data, even when only a small set of labeled data is available. The two experimental data analyses show that the accuracy rates have been improved significantly. Our algorithm can reduce the time needed for the initial training process of BCI systems.
2. By dividing the whole unlabeled data set into several subsets and employing selective learning, the batch-mode incremental learning method significantly reduces the computational burden for training the semisupervised SVM. The data analysis shows that our algorithm is much faster than the one using mixed integer programming directly.
3. The incremental learning characteristic of our algorithm provides us with an online learning algorithm, which is useful for the real-world BCI systems since almost all the real-world BCI systems work online.

Our experimental data analysis shows that our semisupervised algorithm outperforms another commonly used semisupervised algorithm —the full bayes classifier-based self-training algorithm. In fact, in most cases of the data analysis, the full bayes classifier-based self-training algorithm fails to improve the accuracy rate. The reason may be that the dimensionality of the dynamic CSP feature is relatively too high compared with the size of the labeled data set, the generalization performance of the initial full bayes classifier is too poor to predict the labels of the unlabeled data. Consequently, it fails to utilize the information of the unlabeled data to improve its generalization performance. Contrarily, the initial classifier of the semisupervised SVM is SVM which obtains a good generalization performance even in the case of small-labeled data set. This enables the semisupervised SVM to predict the labels of unlabeled data accurately to some extent even when the size of labeled data set is small. Thus, it can successfully utilize the unlabeled data to adjust the parameters of the classifier and further improve its performance.

Although CSP feature is very powerful in discriminating two brain states, a sufficient training data set is needed to determine the transformation matrix. Otherwise, the obtained CSP features and subsequent classification result are not reliable. In this case, our semisupervised learning algorithm may not work. Thus, we suggest a two-stage feature extraction method, that is, we use a dynamic power feature to replace dynamic CSP feature in the first stage of our algorithm, then use the dynamic CSP feature in the second stage of our algorithm. Data analysis results also demonstrate the effectiveness of this method.

## Figures and Tables

**Table 1:** Accuracy rates (%) for the three subjects AA, BB, and CC.

Case	AA	BB	CC	Average
	Accuracy rate	Accuracy rate	Accuracy rate	Accuracy rate
1	94.52	91.84	91.51	92.62
2	89.82	75.53	69.50	78.28
3	97.39	94.47	95.76	95.87
4	—	—	—	—
5	96.08	50.00	50.54	65.54
6	52.48	50.00	50.54	51.01

**Table 2:** CPU time (s) of the three subjects AA, BB, and CC for training the semisupervised SVM.

Case	AA	BB	CC	Average
	Training time	Training time	Training time	Training time
1	1186.40	375.72	568.51	710.21
3	>86400	>86400	>86400	>86400

**Table 3:** Accuracy rates (%) for the independent test set of movement imagination data analysis.

Case	1	2	3	4	5	6
Accuracy rate	89.00	72.00	90.00	—	69.00	69.00

**Table 4:** CPU time (s) for training the semisupervised SVM.

Case	1	4
Training time	233.66	>86400

## Footnotes

The number of elements in a subset can be set according to the performance of the user's computer. If the number of elements in a subset is too small, the classifier will be updated too frequently. In contrast, if the number of elements is too large, the computer cannot solve the problem within an acceptable time period.

Note that only the samples at the time when the user was controlling the cursor were used, that is, 368 samples each trial for subject AA and BB, 304 samples each trial for subject CC; the samples before and after cursor control were omitted.
